# Outcome of patients after lower limb fracture with partial weight bearing postoperatively treated with or without anti-gravity treadmill (alter G®) during six weeks of rehabilitation – a protocol of a prospective randomized trial

**DOI:** 10.1186/s12891-017-1461-0

**Published:** 2017-03-14

**Authors:** Ralf Henkelmann, Sebastian Schneider, Daniel Müller, Ralf Gahr, Christoph Josten, Jörg Böhme

**Affiliations:** 1 Clinic of Orthopaedics, Trauma and Plastic Surgery, University of Leipzig, Liebigstr. 20, Leipzig, 04103 Germany; 2 Hospital St. Georg gGmbH, Clinic of Trauma, Orthopedic and Septic Surgery, Delitzscher Str. 141, Leipzig, 04129 Germany; 3 Ambulantes Reha Centrum, Bauhofstr. 3, Leipzig, 04103 Germany

## Abstract

**Background:**

Partial or complete immobilization leads to different adjustment processes like higher risk of muscle atrophy or a decrease of general performance. The present study is designed to prove efficacy of the anti-gravity treadmill (alter G®) compared to a standard rehabilitation protocol in patients with tibial plateau (group 1)or ankle fractures (group 2) with six weeks of partial weight bearing of 20 kg.

**Methods and Design:**

This prospective randomized study will include a total of 60 patients for each group according to predefined inclusion and exclusion criteria. 1:1 randomization will be performed centrally via fax supported by the Clinical Trial Centre Leipzig (ZKS Leipzig). Patients in the treatment arm will be treated with an anti-gravity treadmill (alter G®) instead of physiotherapy. The protocol is designed parallel to standard physiotherapy with a frequency of two to three times of training with the treadmill per week with duration of 20 min for six weeks.

**Discussion:**

Up to date no published randomized controlled trial with an anti-gravity treadmill is available. The findings of this study can help to modify rehabilitation of patients with partial weight bearing due to their injury or postoperative protocol. It will deliver interesting results if an anti-gravity treadmill is useful in rehabilitation in those patients. Further ongoing studies will identify different indications for an anti-gravity treadmill. Thus, in connection with those studies, a more valid statement regarding safety and efficacy is possible.

**Trial registration:**

NCT02790229 registered on May 29, 2016

## Background

Partial or complete immobilization leads to different adjustment processes like muscle wasting or a decrease of general performance. The degeneration of the immobilized muscle groups and early joint stiffness are essential factors causing a prolonged course of healing [1–4].

An injury with subsequent time of immobilization is a challenge for patients to regain muscle mass and function. Despite animal studies proving that immobilization causes muscle atrophy, until now, it has not been possible to counteract muscle degeneration during immobilization.

To date, only several case reports or small case series dealing with rehabilitation with an anti-gravity treadmill exist. Most of them are cases with individual treatment of one patient [5–7].

Saxena et al. compared patients treated with or without anti-gravity treadmill after surgery for Achilles tendon tears. They included eight patients per group and found no difference between the groups [8].

Two studies by Webber and Hoffman in elderly and young patients showed an improvement of their oxygen exchange with decrease of heart and breathing rate during training in an anti-gravity treadmill. Thus it was possible to perform higher intensity training [9, 10].

A recent study by Peeler et al. used an anti-gravity treadmill for pain reduction and muscle training in patients with knee arthrosis. They were able to prove that an anti-gravity treadmill is easy to use and they could demonstrate with their patients an increase in muscle strength and joint function plus a pain decrease [11].

Cutuk et al. demonstrated no negative effect to blood pressure or heart rate independently of the pressure in the anti-gravity treadmill. Thus they concluded that patients with hypertension or higher cardiovascular risks can be treated with an anti-gravity treadmill. Moreover they showed a positive effect on range of motion of the knee and ankle [12].

Summarizing previous studies demonstrated safety and, to a limited degree, efficacy of treatment with an anti-gravity treadmill during rehabilitation. To our knowledge no prospective randomized study is available in order to prove efficacy of an anti-gravity treadmill compared to a standard rehabilitation protocol.

### Aim of this study

**Fig. 1:**
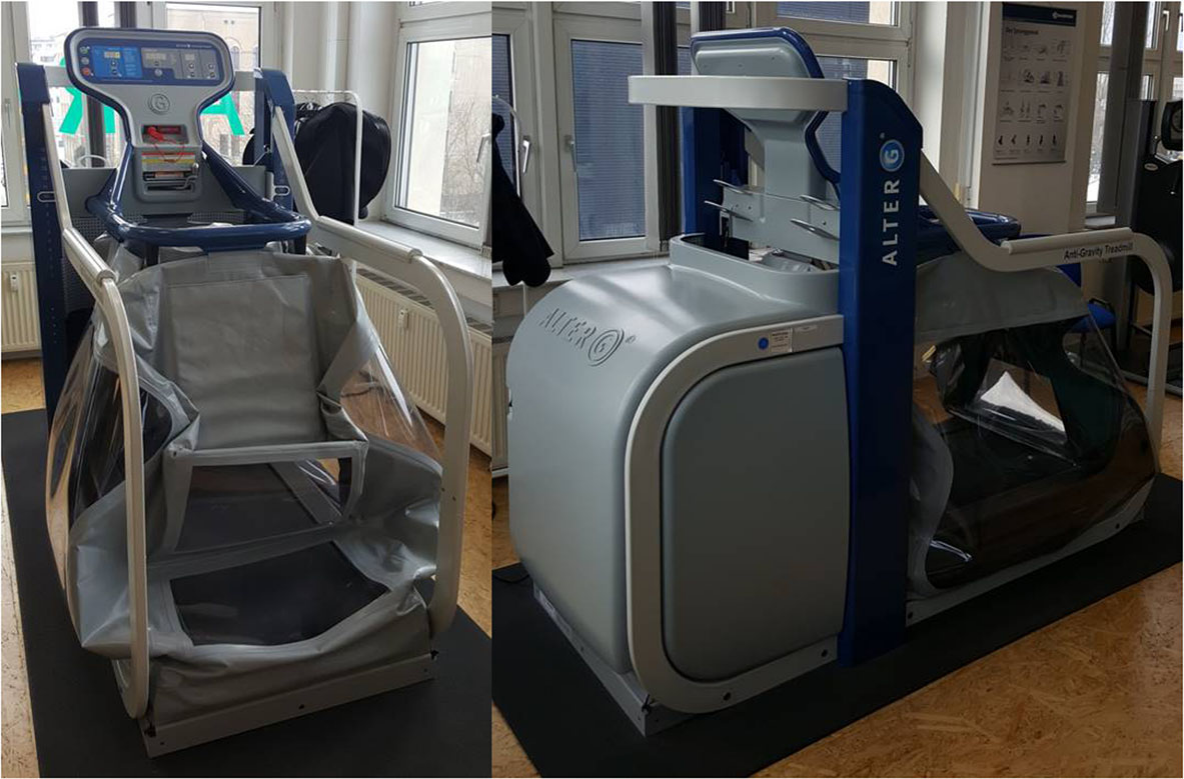
Anti-gravity treadmill (alter G®)

The presented study is designed to prove efficacy of the anti-gravity treadmill (alter G®, Fig. 1) compared to a standard rehabilitation protocol in patients with tibial plateau or ankle fractures with six weeks of partial weight bearing in regard to both subjective and objective variables at predefined times as described under “outcome measures”.

## Methods/Design

### Recruiting hospitals

Two hospitals recruit patients for the study. Both hospitals are national trauma centers. Retrospective data analysis showed that between 80 and 100 patients with ankle or proximal tibia fractures are treated per year in each hospital.

### Participants

The study will include 60 patients per group, both men and non-pregnant women with postoperative partial weight bearing of 20 kg after tibial plateau (group 1) or ankle fracture (group 2). It will include patients aged 18 to 65 years old.

### Inclusion and exclusion criteria

All patients will be assessed clinically preoperatively prior to inclusion to avoid subsequent exclusion. The definitive inclusion will be done after surgery and assignment to partial weight bearing of 20 kg for six weeks. Exclusion criteria are patients with more than 100 kg body weight, restriction of range of motion due to fracture morphology, serious illness or poor general health as judged by a physician that may influence the rehabilitation, open fractures (>1° according to Gustilo and Anderson) or surgical site infection which would cause a longer hospital stay, pregnancy, neuromuscular disorders, or preexisting muscle atrophy. Patients declining participation in the trial (and who fulfill the inclusion criteria) or withdrawing during the study will receive appropriate treatment according to the standard of care.

### Randomization

**Fig. 2:**
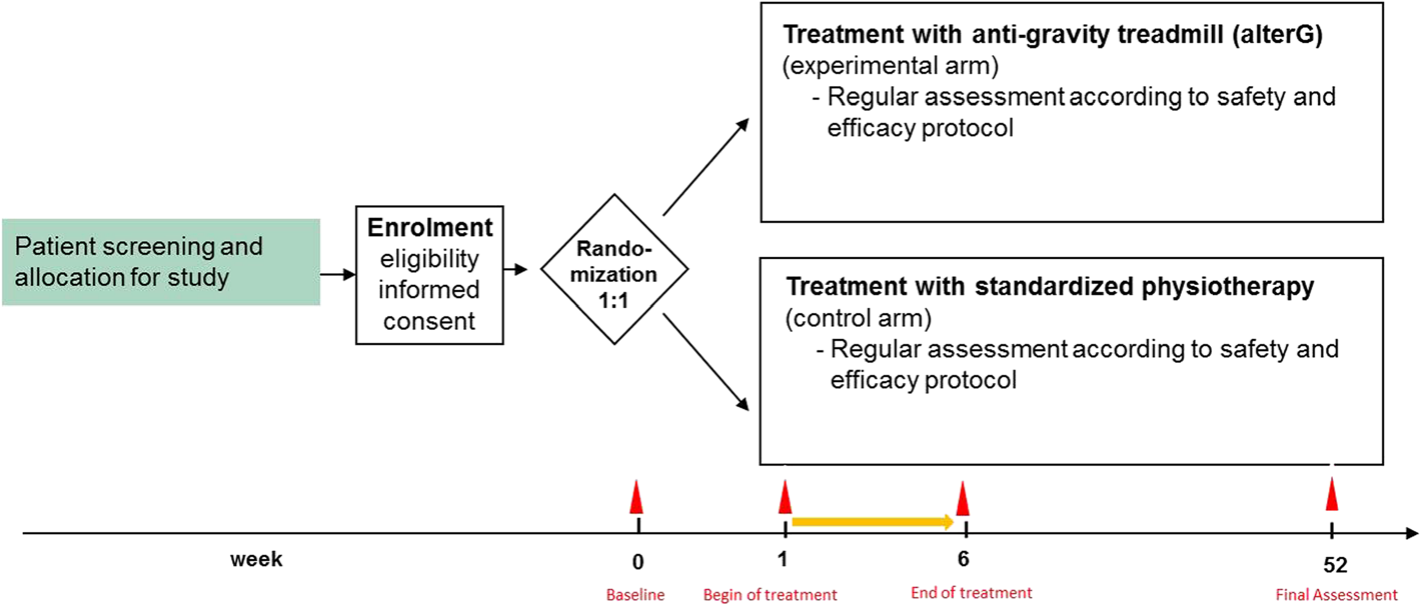
Flow-chart of randomization and study groups

Randomization will be performed for each group centrally via fax supported by the Clinical Trial Centre Leipzig (ZKS Leipzig). Allocation to the experimental arm (randomization ratio 1:1) uses block randomization with variable block lengths (Fig. 2). Randomization will not be stratified.

### Intervention

Treatment of patients with partial body weight bearing of 20 kg after surgery for a tibial plateau fracture (group1) or ankle fracture (group 2) with an anti-gravity treadmill (alter G®) instead of physiotherapy.

### Rehabilitation protocol

During the hospital stay all patients will receive manual lymphatic drainage, cryotherapy and physiotherapy with mobilization under partial weight bearing with 20 kg of the affected leg with the aim of pain reduction and to gain mobility. Patients in both groups will be treated with full range of motion of the affected joint without limitation.

Patients in the experimental arm will be treated with manual lymphatic drainage, cryotherapy and a fixed protocol of training in an anti-gravity treadmill (alter G®, Table 1) two to three times a week for six weeks. The anti-gravity treadmill consists of a treadmill with a surrounding chamber. A seal is achieved between the patient and the chamber through the use of a neoprene kayak-type skirt that fastens over a lip in the aperture opening of the chamber. The pressure inside the chamber is increased above the external pressure with an air compressor. In this way patients can be treated on the treadmill under simulated fractional gravity.

**Table 1:** Protocol of training in the anti-gravity treadmill (alter G®)

Week	Duration per visit	Speed	Partial body weight	Grade incline (degrees)
1 + 2	20 min	5 min @ 1.5 km/h 5 min @ 2 km/h 5 min @ 1.5 km/h 5 min @ 2 km/h	20 kg	1
3 + 4	20 min	5 min @ 3 km/h 5 min @ 2 km/h 5 min @ 3 km/h 5 min @ 2 km/h	20 kg	1
5	20 min	5 min @ 4 km/h 5 min @ 3.5 km/h 5 min @ 4 km/h 5 min @ 3.5 km/h	20 kg	1
6	20 min	8 min @ 4 km/h 2 min @ 5 km/h 8 min @ 4 km/h 2 min @ 5 km/h	20 kg	1

The protocol is designed parallel to standard physiotherapy with a frequency of two to three times a week and a duration of 20 min for six weeks. Cryotherapy and lymphatic drainage will be performed for 20 min until swelling of the soft tissue of the affected region will be disappeared.

Patients in the control arm will be treated according to clinic standard with manual lymphatic drainage, cryotherapy and 20 min of physiotherapy two to three times a week for six weeks. Standard physiotherapy includes training with a continuous passive motion machine, physical therapy with coordination, balance and motion training. Physiotherapy will be done according to a standardized protocol [13]. Cryotherapy and lymphatic drainage will be performed for 20 min until swelling of the soft tissue of the affected region will be disappeared.

## Outcome measures

### Demographics

Demographics to be collected at inclusion include age, gender, height (cm), weight (kg), body mass index (BMI), injury mechanism, previous medical history, current medication, smoking or drug/alcohol abuse, social status, work status and health insurance.

### Primary endpoint

Change of overall KOOS (Knee Injury and Osteoarthritis Outcome Score; group 1) or FOAS (Foot and Ankle Outcome Score; group 2) from baseline (Day 1 after operation) to Final Assessment (FA) 12 months after operation determined for each group and between the groups [14–18].

The KOOS or FAOS are patient-reported outcome measurement instruments. They are widely used in clinical trials, and its psychometric properties have been validated. They are available in a validated German version.

The KOOS or FAOS consist of five separately scored and validated subscales: Pain, Symptoms, Function in daily living (ADL), Function in Sport and Recreation (Sport/Rec), and knee/ankle related Quality of Life (QOL). The scores scale all range from 0 (worst outcome) to 100 (best outcome). For purpose of clinical trials, it has been recommended to create a composite score, by averaging the subscale scores of interest in the respective trial.

For further information see www.koos.nu.

### Secondary endpoint

Change of the 5 subscores of the KOOS or FAOS (Pain, other Symptoms, Function in daily living (ADL), Function in sport and recreation (Sport/Rec), knee/ankle related Quality of life (QoL)) from baseline (Day 1 after operation) to 6 and 12 weeks and 12 months after operation determined for each group and between the groups.

Circumference measurement of tights and lower leg on both legs 10 cm and 20 cm above the knee joint space and 10 cm below (knee in neutral position) from baseline (Day 1 after surgery) to day of discharge (7-11d), 3, 6 and 12 weeks and 12 months final assessment after surgery determined for each group and between the groups [3].

Range of motion (ROM) of the affected joint determined by neutral zero method from baseline (Day 1 after operation) to 6 and 12 weeks and 12 months after operation determined for each group and between the groups.

Short Form Health Survey (SF-36) from baseline (Day 1 after operation) to 6 and 12 weeks and 12 months after operation determined for each group and between the groups [19].

Dynamic gait index (DGI) from day of discharge (7-11d to 3, 6 and 12 weeks and 12 months after operation determined for each group and between the groups [20].

### Tertiary endpoint

Number of days of absence from work (employment) and/or days of inability to follow usual activities until final assessment and time point when patient was back to work and/or to follow usual activities. Data will be recorded for each group and compared between the groups.

### Hypothesis

Null hypothesis: There is no difference in overall KOOS (group 1) or FAOS (group 2) following rehabilitation with or without an anti-gravity treadmill (alter G®) 12 months after surgery.

### Statistical analysis

Demographic and clinical characteristics will be presented as means and standard deviations (SD) or frequencies and percentages, as appropriate. The normality of continuous data will be assessed by examining the histograms. If necessary, a suitable transformation will be considered to symmetrize the data.

Our primary efficacy endpoint for tibial plateau fractures/ankle fractures is change from baseline (first day postoperative) to 12 months in overall KOOS/FOAS. Literature data suggest a standard deviation of about 18 in KOOS4/FOAS. We expect a similar standard deviation for overall KOOS/FOAS. The developer of the KOOS/FOAS reports a minimally important change of 8–10 points.

We want to power this study to detect a moderate effect of 15 points using a two-sided *t*-test with a significance level of 0.05. With 25 patients per arm per group, we will have a power of 82% to detect a difference between groups of 15 points (48% power for a difference of 10). Using a one-sided *t*-test, the power figures are 89% (resp. 61%).

### Sample size

According to statistical analysis of the primary endpoint following sample size was set.

To be allocated to trial (*n =* 120; group 1: *n =* 60 tibial plateau fractures, group 2: *n =* 60 ankle fractures)

To be analysed (*n =* 100; *n =* 50 tibial plateau fractures, *n =* 50 ankle fractures)

### Risk assessment

Parallel to the study protocol a safety protocol was established. All patients will be examined pre-operatively for inclusion and during the hospital stay on the day after surgery and the day of discharge. Furthermore, clinical examinations will be done in the outpatient clinic after 3, 6 and 12 weeks and to FA (Table 2).

**Table 2:** Summary of safety and efficacy study protocol

Summary of safety and study protocol								
		pre-operative entry examination	during hospital stay		out-patient clinic			
								FA
		0d	+1d	+7–11d	" + 3 we	+6we	+12 we	+12 mo
Safety	S1	x	x	x	x	x	x	x
	S2				x	x		
Efficacy	E1	x				x	x	x
	E2	x		x	x	x	x	x
	E3	x				x	x	x
	E4			x	x	x	x	x
	E5							x
S 1 = clinical examination			E 1 = KOOS/FOAS					
S 2 = interview before and after alterG training			E 2 = measuring circumference and joint mobility with neutral zero method					
			E 3 = SF −36					
			E 4 = Dynamic Gait Index (DGI)					
			E 5 = days of absence from work/registration of vocational retraining					

d = day, wk = week, mo = month, FA = final assessment

All adverse or severe adverse events will be recorded. So far, with the anti-gravity treadmill, no adverse events or severe adverse events have been described. After every training patient will be interviewed by the physiotherapist and heart rate, blood pressure, body weight, numeric rating scale (NRS), pain medication and general condition will be documented.

## Discussion

To date, no published randomized controlled trial with an anti-gravity treadmill is available. Two ongoing studies can be found for postoperative indications (NCT01689922, NCT02628002) and one dealing with the topic of postoperative treatment after total knee arthroplasty (NCT02426190). A recent study by McNeill et al. stated that there are significant differences between reported and measured body weight support on the anti-gravity treadmill [21]. Thus it could be possible that during training in the treadmill with 20 kg body weight partial loading might be higher than 20 kg. Several studies showed that the majority of patients could not reproduce accurately partial weight bearing and exceeded their target load with about 20 kg over target load [22–24]. Hence we accepted a possible inaccuracy of the treadmill since it is known that patients cannot put partial weight bearing of 20 kg into practice. Furthermore, an unintentional overload by the patient while walking with crutches is possible and unavoidable in both groups. A possible variance due to inaccuracy of the antigravity treadmill could be present.

The findings of this study can help to modify the rehabilitation of patients with partial weight bearing due to their injury or postoperative protocol. It will deliver interesting results if an anti-gravity treadmill is useful in rehabilitation in those patients. Further ongoing studies will prove different indications for an anti-gravity treadmill. Thus in connection with those studies a more valid statement due to safety and efficacy is possible.
